# Predictors of persistently positive Mycobacterium-tuberculosis-specific interferon-gamma responses in the serial testing of health care workers

**DOI:** 10.1186/1471-2334-10-220

**Published:** 2010-07-23

**Authors:** Felix C Ringshausen, Albert Nienhaus, Anja Schablon, Stephan Schlösser, Gerhard Schultze-Werninghaus, Gernot Rohde

**Affiliations:** 1 Department of Medicine III - Pneumology, Allergology and Sleep Medicine, University Hospital Bergmannsheil, Bochum, Germany; 2 Department of Medicine, Spital Bülach, Bülach, Switzerland; 3 Department of Occupational Health Research, Institution for Statutory Accident Insurance and Prevention in Health and Welfare Services, Hamburg, Germany; 4 Department of Occupational Medicine, University Hospital Bergmannsheil, Bochum, Germany; 5 Department of Respiratory Medicine, Maastricht University Medical Centre, Maastricht, The Netherlands

## Abstract

**Background:**

Data on the performance of Mycobacterium-tuberculosis-specific interferon-(IFN)-γ release assays (IGRAs) in the serial testing of health care workers (HCWs) is limited. The objective of the present study was to determine the frequency of IGRA conversions and reversions and to identify predictors of persistent IGRA positivity among serially tested German HCWs in the absence of recent extensive tuberculosis (TB) exposure.

**Methods:**

In this observational cohort-study HCWs were prospectively recruited within occupational safety and health measures and underwent a tuberculin skin test (TST) and the IGRA QuantiFERON^®^-TB Gold In-Tube (QFT-GIT) at baseline. The QFT-GIT was repeated 18 weeks later in the median. QFT-GIT conversions (and reversions) were defined as baseline IFN-γ < 0.35 IU/ml and follow-up IFN-γ ≥ 0.35 IU/ml (and vice versa). Predictors of persistently positive QFT-GIT results were calculated by logistic regression analysis.

**Results:**

In total, 18 (9.9%) and 15 (8.2%) of 182 analyzed HCWs were QFT-GIT-positive at baseline and at follow-up, respectively. We observed a strong overall agreement between baseline and follow-up QFT-GIT results (κ = 0.70). Reversions (6/18, 33.3%) occurred more frequently than conversions (3/162, 1.9%). Age and positive prior and recent TST results independently predicted persistent QFT-GIT positivity. Furthermore, the chance of having persistently positive QFT-GIT results raised about 3% with each additional 0.1 IU/ml increase in the baseline IFN-γ response (adjusted odds ratio 1.03, 95% confidence interval 1.01-1.04). No active TB cases were detected within an observational period of more than two years.

**Conclusions:**

The QFT-GIT's utility for the application in serial testing was limited by a substantial proportion of reversions. This shortcoming could be overcome by the implementation of a borderline zone for the interpretation of QFT-GIT results. However, further studies are needed to clearly define the within-subject variability of the QFT-GIT and to confirm that increasing age, concordantly positive TST results, and the extend of baseline IFN-γ responses may predict the persistence of QFT-GIT positivity over time in serially tested HCWs with only a low or medium TB screening risk in a TB low-incidence setting.

## Background

In high-income countries with a low burden of tuberculosis (TB) targeted screening of at-risk groups, identification and preventive treatment of latent TB infection (LTBI, i. e. lasting Mycobacterium-tuberculosis-[MTB]-specific T-cell responses in the absence of clinical evidence for TB disease) in individuals with recent exposure are fundamental components of TB control strategies [1,2]. Health care workers (HCWs) are considered at risk for the occupational transmission of TB infection due to nosocomial exposure [3,4]. Hence, screening procedures within occupational safety and health (OSH) measures, i. e. contact investigations after exposure to TB source cases as well as the serial testing of HCWs with continuous risk for workplace exposure, are required for TB surveillance and with regard to the recognition and compensation of TB as an occupational disease.

The use of the tuberculin skin test (TST) as a tool for serial testing is limited by cross-reactivity following Bacillus Calmette-Guérin (BCG) vaccination and exposure to non-tuberculous mycobacteria, nonspecific variations, and boosting [5]. In this respect, novel interferon-(IFN)-γ release assays (IGRAs) provide distinct advantages. They are highly MTB-specific and thus not confounded in populations containing a high proportion of BCG-vaccinated individuals. They avoid boosting of immune responses by ex-vivo testing and possess logistical conveniences [6,7]. Although broadly recommended and increasingly used [8,9], data on the interpretation of IGRA results in serial testing is scarce. A limited number of studies are available regarding the performance of IGRAs in serial testing in high-burden countries [10-12], the effect of treatment of active TB or LTBI on IGRA responses [13-17], and the within-subject variability (reproducibility) over different periods of time [18-20]. Only few studies cover their use in HCWs in intermediate and high burden countries [7,21,22] or general populations in high-income, low-incidence countries [13,23-25].

Until now, no published study evaluated the performance of an IGRA in the serial testing of HCWs in a TB low-incidence country like Germany, where the annual TB incidence rate was 6.1 per 100,000 population in 2007 [26]. We hypothesized that the risk of progression to active TB among German HCWs with persistently positive IGRA results, but without recent extensive exposure is low, and that the repeated IGRA testing within OSH screening measures may not have an additive value among those subjects. In this context, the identification of predictors of persistent IGRA positivity could contribute to the restriction of IGRA serial testing and thus to the reduction of costs.

It was the aim of the present study to evaluate the performance of the IGRA QuantiFERON^®^-TB Gold In-Tube (QFT-GIT) in the serial testing of German HCWs in the absence of recent extensive exposure, to determine the frequency of inconsistent QFT-GIT results (conversions and reversions), and to identify independent predictors of persistent QFT-GIT positivity.

## Methods

### Study design and subjects

In this prospective, observational cohort-study we enrolled eligible HCWs between December 2005 and January 2008 at three German hospitals (Bochum, Großhansdorf, Hamburg). All HCWs were subject to screening procedures according to German OSH legislation and were classified to low or medium TB screening risk according to the Centers for Disease Control and Prevention (CDC) guidelines [27].

The majority of enrolled HCWs (149/197, 75.6%) were recruited from TB contact investigations after limited exposure to culture-confirmed TB source cases. As there was no evidence of ongoing transmission among those HCWs, all had been classified as medium TB screening risk after the baseline evaluation and had been referred to OSH screening for follow-up. One hundred and forty-four HCWs had been exposed to a single smear-negative TB source case with low contagiosity [28], one HCW had been exposed to another smear-negative source case, and four HCWs had had limited contact ≤ 8 hours to a smear-positive source case.

All subjects were evaluated at baseline using a standardized interview and questionnaire, a one-step Mantoux TST (two tuberculin units, 0.1 ml purified protein derivate [PPD] RT 23, Statens Serum Institute, Copenhagen, Denmark), the IGRA QFT-GIT (Cellestis, Carnegie, Australia), and chest x-ray if the baseline QFT-GIT results were positive or showed a conversion at follow-up. The follow-up included a second QFT-GIT only. The subsequent suggestion for a preventive chemotherapy according to current national and international recommendations [1,8], as well as the determination regarding the interval between both IGRAs were the responsibility of the respective occupational physician. The latter mainly depended on the underlying screening risk classification, and the fact whether the respective HCW was screened according to the "infection protection act", or the "biological agents regulation" of the German OSH legislation, i. e. if the follow-up QFT-GIT was performed rather short-term after participation in a recent contact investigation (e. g. 3-6 months after the baseline evaluation), or if the HCW was subject to (bi-)annual routine screening according to his workplace risk (e. g. someone working in ID/TB care without recent exposure).

Inclusion criteria were an age of 18 years and above, engagement in health care during the study period, and both valid baseline TST and QFT-GIT as well as follow-up QFT-GIT results. Individuals who were recruited from a setting with evidence of ongoing transmission or who had been exposed to smear-positive TB > 8 hours were excluded. The study cohort was longitudinally observed regarding the progression to active TB for a period of more than two years until February 7^th ^2010 (32 months in the median). All HCWs were followed up according to German OSH legislation. QFT-GIT-positive subjects were radiologically followed up as recommended by national guidelines [8].

### Diagnostic methods

The questionnaire, the evaluation of exposure, the application of the TST, and the performance of the QFT-GIT have been described previously [28]. The BCG vaccination status was reassured by medical records or the presence of vaccination scars. The baseline TST and QFT-GIT were performed simultaneously. TST indurations > 5 mm and ≥ 10 mm, respectively, were considered positive according to the respective clinical situation, the TB screening risk classification, and national and international guidelines [8,27]. The occupational physicians who read the TST were blinded to the QFT-GIT results determined by the laboratory team and vice versa. The QFT-GIT was performed according to the manufacturer's instructions that consider a result positive if the IFN-γ response of TB antigen minus Nil was ≥ 0.35 IU/ml [29]. Conversion was defined as a baseline IFN-γ concentration < 0.35 IU/ml and a follow-up IFN-γ concentration ≥ 0.35 IU/ml. Reversion was defined vice versa. All IGRAs were retested at the same center as the baseline IGRA.

### Statistical analysis

Data analysis was performed using SPSS, version 11.5 (SPSS Inc., Chicago, Illinois). Categorical data were compared by Pearson's chi-squared or Fisher's exact test, where appropriate. Normal distribution in continuous variables was determined with the Kolmogorov-Smirnov test and differences were subsequently determined either with the paired student's t-test, the Mann-Whitney-U-test, or the Wilcoxon test. Spearman correlation coefficients and kappa values were calculated for both tests. Independent predictors of persistent QFT-GIT positivity were identified using logistic regression. All potential predictors or confounders of interest were entered simultaneously and model building was performed backward using the chance criteria for variable selection. Variables considered to be clinically significant were retained regardless of statistical significance [30]. Relations were described as adjusted odds ratio (OR) and 95% confidence interval (CI), with significance assessed by p-values computed from Wald statistics. All p-values reported were calculated two-tailed with statistical significance set to p ≤ 0.05. The study protocol was approved by the ethics committee of the Hamburg Medical Council and the Ruhr-University Bochum. All study participants gave their written and informed consent.

## Results

### Study population

**Figure 1:**
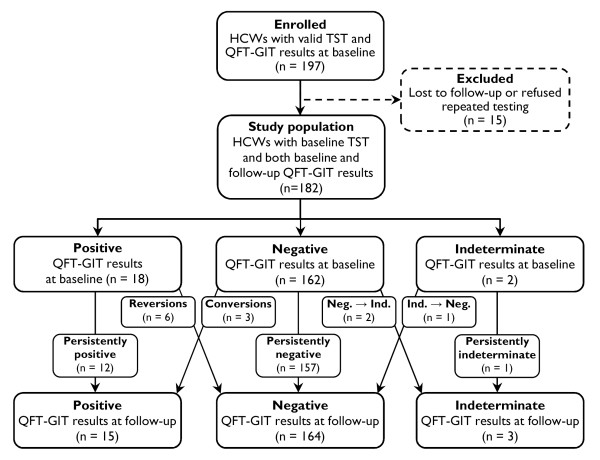
**Study flow chart with baseline and follow-up QFT-GIT results**. HCWs = health care workers; QFT-GIT = QuantiFERON^®^-TB Gold In-Tube; TST = tuberculin skin test.

One hundred and ninety-seven HCWs were enrolled in the present study. Fifteen subjects (with negative QFT-GIT results) were lost to follow-up or refused repeated testing. Finally, 182 HCWs (92.4%) constituted the study population (Figure 1). The demographic and clinical features of the study population are shown in Table 1. The median interval between both QFT-GIT was 18 weeks (range 11-105 weeks). The mean age was 38 ± 10 years (range 19-62) and the mean duration of employment in health care was 14 ± 10 years (range 1-42). As these variables were highly correlated (r = 0.73, p < 0.0001), the latter was not considered for further analysis. The majority of subjects included in the final study population were recruited from TB contact investigations (134/182, 73.6%). Of those, 129 HCWs (96.3%) had been exposed to a single smear-negative source case. The median cumulative exposure time among all subjects recruited from contact investigations was 1.0 hour (range 3 minutes to 67 hours). Exposure > 40 hours occurred in four individuals only, who had all been exposed to smear-negative TB. However, exposure time had no significant impact on the subsequent test results among this subpopulation (see additional file 1: Influence of exposure to TB source cases on subsequent test results, which also provides a detailed description of the four HCWs that had been exposed to smear-positive TB source cases).

**Table 1:** Characteristics of the study population

Variables	n	%
Subjects, total	182	100
TB screening risk classification		
Low risk	17	9.3
Medium risk	165	90.7
Reason for serial testing		
(Bi-)Annual routine screening	48	26.4
Follow-up after recent contact investigation	134	73.6
Sex		
Male	59	29.1
Female	123	70.9
Age categorized		
18 to 39 years	104	57.1
40 to 49 years	50	27.5
≥ 50 years	28	15.4
Foreign country of birth*	40	22.0
Birth in high-burden country^#^	8	4.4
BCG vaccination		
Yes	95	52.2
No	75	41.2
Unknown	12	6.6
Health care professions		
Nursing	56	30.8
Physician	28	15.4
Other	98	53.8
Affiliation with ID/TB Care	43	23.6
Family history of TB	14	7.7
Own history of TB	2	1.1
Prior TST	129	70.9
TST results		
Positive prior TST result	54	41.9
Recent TST > 5 mm induration	52	28.6
Recent TST ≥ 10 mm induration	44	24.2
QFT-GIT results at baseline		
Positive	18	9.9
Negative	162	89.0
Indeterminate	2	1.1
QFT-GIT results at follow-up		
Positive	15	8.2
Negative	164	90.1
Indeterminate	3	1.7

*Mostly Turkey (n = 17) and Poland (n = 9). ^#^TB high-burden countries (according to WHO): Belarus (n = 2), Bosnia and Herzegovina (n = 1), Kazakhstan (n = 2), Morocco (n = 2), Philippines (n = 1). BCG = Bacillus Calmette-Guérin; ID = Infectious Diseases. QFT-GIT = QuantiFERON^®^-TB Gold In-Tube. TB = tuberculosis; TST = tuberculin skin test.

### Prior and baseline TST results

The prior and the recent TST results are shown in Table 1. The TST was administered at baseline only. One hundred and twenty-nine subjects (70.9%) had been tested with a prior TST, in the median 5 years ago (range 3 months to 38 years). In most instances, the prior TST had been administered by the qualitative multi-puncture method (115/129, 89.1%) and showed no significant effect on the baseline TST and QFT-GIT results (see additional file 2: Table S2 - Agreement and time interval between prior and baseline TST and QFT-GIT results).

### QFT-GIT results and agreement with recent TST results

The QFT-GIT results at baseline and at follow-up are shown in Table 1 and Figure 1. The total frequency of indeterminate QFT-GIT results was 1.4% (5 of all 364 performed IGRAs). Overall, the agreement between the TST (> 5 mm cut-off) and baseline QFT-GIT results was low (raw 72.5%, κ = 0.17, p = 0.012), and only slightly better with follow-up QFT-GIT results (raw 75.3%, κ = 0.23, p < 0.001). At best, a moderate agreement was observed among non-BCG-vaccinated subjects between the TST (≥ 10 mm cut-off) and the baseline QFT-GIT (raw 89.2%, κ = 0.50, p < 0.001). Subjects with positive baseline QFT-GIT results had significantly higher baseline IFN-γ levels when they were concordantly TST-positive (≥ 10 mm cut-off, median 4.33 vs. 1.26 IU/ml, p = 0.001).

### Consistency between baseline and follow-up QFT-GIT results

**Figure 2:**
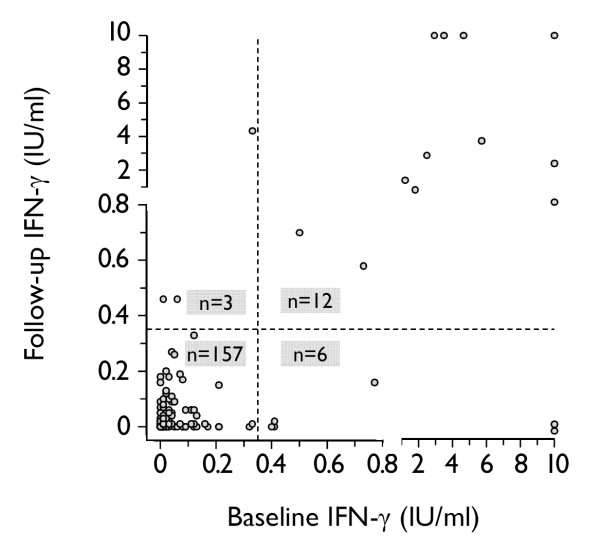
**Distribution of IFN-γ responses in the baseline and the follow-up QFT-GIT**. The responses of four subjects with indeterminate QFT-GIT results are not shown. The vertical and the horizontal dashed lines represent the QFT-GIT's diagnostic cut-off of 0.35 IU/ml. IFN-γ responses ≥ 10 IU/ml are shown as 10 IU/ml, IFN-γ responses < 0.00 IU/ml are shown as 0.00 IU/ml. IFN = interferon; QFT-GIT = QuantiFERON^®^-TB Gold In-Tube.

Overall, 170 of 182 subjects (93.4%) had consistent QFT-GIT results. Figure 2 shows the distribution of IFN-γ responses for positive and negative QFT-GIT results at baseline and at follow-up. There was a strong overall agreement between both QFT-GIT results (κ = 0.70, p < 0.0001). The agreement between both QFT-GIT results stratified according to prior and recent TST results is shown in Table 2.

**Table 2:** Consistency between baseline and follow-up QFT-GIT stratified by TST

		Follow-up QFT-GIT		
			
	Baseline QFT-GIT	Positive (n = 15) n (%)	Negative (n = 163) n (%)	Agreement
All subjects* (n = 178)	Positive (n = 18)	12 (6.7)	6 (3.4)	Raw = 94.9% κ = 0.70^#^
	Negative (n = 160)	3 (1.7)	157 (88.2)	
Positive prior TST (n = 54)	Positive (n = 9)	9 (16.7)	0	Raw = 100% κ = 1.0^#^
	Negative (n = 45)	0	45 (83.3)	
Recent TST > 5 mm (n = 51)	Positive (n = 10)	9 (17.6)	1 (2.0)	Raw = 94.1% κ = 0.82^#^
	Negative (n = 41)	2 (3.9)	39 (76.5)	
Recent TST ≥ 10 mm (n = 44)	Positive (n = 10)	9 (20.5)	1 (2.3)	Raw = 93.2% κ = 0.81^#^
	Negative (n = 34)	2 (4.5)	32 (72.7)	

*Four individuals with indeterminate QFT-GIT results were excluded from this analysis. ^#^p < 0.0001, each. QFT-GIT = QuantiFERON^®^-TB Gold In-Tube. TST = tuberculin skin test.

### Frequency of inconsistent QFT-GIT results

Inconsistent QFT-GIT results occurred in 12 of 182 HCWs (6.6%), and thus were comparatively rare even when using a simplistic dichotomous negative-to-positive approach. The characteristics of individuals with inconsistent QFT-GIT results are shown in Table 3. Three of 162 subjects had conversions (1.9%), and six of 18 subjects had reversions (33.3%, Figure 1, Figure 2). Another three subjects had inconsistent results including indeterminate QFT-GIT results (a change from negative to indeterminate and vice versa, Figure 1). Notably, a significant proportion of conversions and reversions occurred around the manufacturer's predefined cut-off (Table 3).

**Table 3:** Characteristics of the subjects with inconsistent QFT-GIT results (n = 12)

							TST results		IFN-γ (IU/ml)		
								
**ID No**.	Age	Sex	Country of birth	Family history of TB (year)	Profession (department/center) - exposure or risk classification	BCG vaccination	Prior TST*	Recent TST (mm)	Baseline	Follow-up	IGRA time interval (weeks)
Conversions^# ^(n = 3)											

079	54	F	Germany	No	Room cleaning (B) - Contact tracing (SNCP, 90 min)^§^	Yes	No	10	0.06	0.46	17
151	39	F	Germany	1967	Administration (Pulmonary/G) - Routine screening (medium risk)	No	No	0	0.01	0.46	52
177	50	F	Germany	1990	Nursing (ER/G) - Routine screening (medium risk)	Yes	Neg. (45)	10	0.26	4.33	54
Reversions^# ^(n = 6)											

008	30	F	Turkey	No	Room cleaning (B) - Contact tracing (SNCP, 20 min)^§^	Unknown	No	0	22.66	0.01	17
015	38	M	Germany	No	Nursing (Surgery/B) - Contact tracing (SNCP, 44 h)^§^	Yes	Neg. (52)	0	0.41	0.00	19
072	54	F	Germany	1955	Nursing (Surgery/B) - Contact tracing (SNCP, 4 h)^§^	No	Neg. (5)	10	0.77	0.16	18
095	39	M	Germany	No	Physical therapy (Rehabilitation/B) - Contact tracing (SNCP, 4 h)^§^	Yes	Neg. (57)	0	29.34	0.00	16
124	44	F	Germany	No	Physical therapy (Rehabilitation/B) - Contact tracing (SNCP, 3 h)^§^	Yes	Neg. (86)	0	0.41	0.00	17
265	26	F	Germany	No	Administration (ER/H) - Contact tracing (SPCP, 8 h)^§^	No	No	0	0.40	0.00	22
Discordantly indeterminate (n = 3)											

086	50	F	Germany	No	Nursing (Surgery/B) - Contact tracing (SNCP, 9 h)^§^	No	Pos. (37)	0	0.00	0.00^†^	17
173	20	F	Germany	No	Nursing (Pulmonary/G) - Routine screening (medium risk)	Yes	No	7	0.01^†^	0.01	51
187	26	F	Germany	No	Nursing (Surgery/B) - Contact tracing (SNCP, 67 h)^§^	Yes	Neg. (60)	0	0.02	0.03^†^	21

*The interval between the prior and the recent TST in months is indicated in parentheses. ^#^QFT-GIT conversions are defined as baseline IFN-γ < 0.35 IU/ml and follow-up IFN-γ ≥ 0.35 IU/ml, QFT-GIT reversions are defined vice versa. ^§^The mean interval between the last exposure to the source case and the baseline evaluation was 17 weeks in individuals initially included in contact investigations. ^†^Indeterminate QFT-GIT result due to insufficient IFN-γ mitogen response. B = Bochum; BCG = Bacillus Calmette-Guérin; ER = emergency room; F = female; G = Großhansdorf; H = Hamburg; IFN = interferon; M = male; QFT-GIT = QuantiFERON^®^-TB Gold In-Tube; SNCP = smear-negative, culture-positive (tuberculosis source case); SPCP = smear-positive, culture-positive (tuberculosis source case); TST = tuberculin skin test.

### Impact of age, TST induration and baseline IFN-γ concentration on follow-up QFT-GIT results

**Figure 3:**
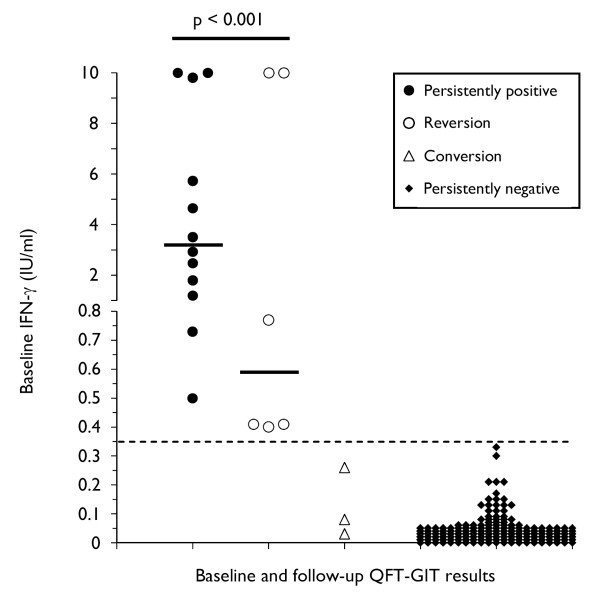
**Comparison of the baseline level of IFN-γ responses and consistency of QFT-GIT results**. The short bar represents the median level of IFN-γ responses in subjects with positive baseline results (3.22 vs. 0.59 IU/ml, p < 0.001). The baseline IFN-γ responses of the subjects with conversions and consistently negative results are plotted for comparison. The responses of four subjects with indeterminate results are not shown. The dashed line represents the diagnostic cut-off ≥ 0.35 IU/ml. IFN-γ responses ≥ 10 IU/ml are shown as 10 IU/ml, IFN-γ responses < 0.00 IU/ml are shown as 0.00 IU/ml. IFN = interferon; QFT-GIT = QuantiFERON^®^-TB Gold In-Tube.

Subjects with persistently positive QFT-GIT results were older (median age 52 vs. 38 years, p < 0.001), had larger TST indurations (median 13 vs. 0 mm, p = 0.006), and had higher IFN-γ responses at baseline compared to subjects with reversions at follow-up (median 3.22 vs. 0.59 IU/ml, p < 0.001, Figure 3).

### Predictors of persistent QFT-GIT positivity

Multiple logistic regression analysis demonstrated that the chance of persistent QFT-GIT positivity increased with advancing age, a positive prior TST result, and a recent TST result > 5 mm and ≥ 10 mm induration, respectively. Furthermore, each additional 0.1 IU/ml increase in the baseline IFN-γ response raised the likelihood of having persistently positive QFT-GIT results by 3% (OR 1.03, 95% CI, 1.01-1.04, Table 4).

**Table 4:** Predictors of persistent QFT-GIT positivity

	Positive Baseline and follow-up QFT-GIT	
	
Variables	Adjusted OR* (95% CI)	p-value
Age categorized^#^	**5.0 (3.9-13.5)**	**0.001**
Sex		
Female	1	-
Male	1.2 (0.2-6.3)	0.80
Birth in high-burden country		
No	1	-
Yes	0.2 (0.0-3.6)	0.29
BCG status	1	-
Vaccinated	0.3 (0.1-1.5)	0.15
Unknown	0.6 (0.0-9.8)	0.70
Profession		
Other	1	-
Nursing	0.6 (0.1-3.4)	0.57
Physician	3.2 (0.5-22.7)	0.24
Affiliation with ID/TB Care		
No	1	-
Yes	0.8 (0.1-7.8)	0.85
Family history of TB		
No	1	-
Yes	1.1 (0.1-8.7)	0.96
QFT-GIT test interval per day	1.0 (0.99-1.01)	0.49
Exposure per hour^§^	0.7 (0.45-1.13)	0.15
Prior TST		
Negative	1	-
Positive	**14.2 (1.4-143.2)**	**0.024**
Recent TST		
Negative	1	-
> 5 mm induration	**12.1 (2.4-59.8)**	**0.002**
≥ 10 mm induration	**15.5 (3.1-81.3)**	**0.001**
Baseline IFN-γ increase per 0.1 IU/ml	**1.03 (1.01-1.04)**	**0.0006**

*Variables included in final model building were age, birth in high-burden country, BCG status, recent TST result > 5 mm or ≥ 10 mm induration, and baseline IFN-γ increase per 0.1 IU/ml. ^#^Compare Table 1. ^§^Exposure per hour in those 134 study subjects that were recruited from contact investigations. BCG = Bacillus Calmette-Guérin; CI = confidence interval; ID = Infectious Diseases; IFN = interferon; OR = odds ratio; QFT-GIT = QuantiFERON^®^-TB Gold In-Tube; TB = tuberculosis; TST = tuberculin skin test.

### Clinical outcome and follow-up

Active TB was ruled out by chest X-ray in all participants with positive baseline QFT-GIT results and those with conversions. None of the 182 study subjects developed active TB within the observational period of more than two years (32 months in the median, range 25-50).

## Discussion

To our knowledge, the present study is the first study, which evaluated the use of an IGRA in the serial testing of HCWs in a TB low-incidence country, and moreover, systematically analyzed predictors of consistent IGRA results. We observed a strong overall agreement between baseline and follow-up QFT-GIT results that further improved in subjects with positive prior and recent TST results. However, reversions occurred in about one third of initially QFT-GIT-positive study subjects. Persistent QFT-GIT positivity was independently predicted by age, prior and recent TST results, and the extent of baseline IFN-γ responses over a median interval of 18 weeks.

### Comparison with previous literature in the field

We found a similarly low rate of positive QFT-GIT results at baseline (9.9%) and follow-up (8.2%) compared to recent TST results (28.6% with a threshold > 5 mm and 24.2% with a threshold ≥ 10 mm). In accordance with studies among HCWs in comparable epidemiological settings, we observed a poor overall agreement between IGRA and TST results due to BCG vaccination in the majority of individuals [31-33]. Even with simplistic negative-to-positive (and vice versa) definitions, and moreover, considering indeterminate IGRA results we found a comparatively low overall frequency of inconsistent IGRA results of 6.6% (12/182). In line with previous studies among HCWs, we observed more reversions (6/18, 33.3%) than conversions (3/162, 1.9%). In a recent study among 311 Japanese HCWs the IGRA conversion rate was comparable to the one we determined (1.8%), while the reversion rate was slightly higher (41%) [22]. Another study from Singapore, an intermediate-incidence country, retested IGRA-negative junior physicians only and described a conversion rate of 4.9% (9/182) [21]. Pai and colleagues reported conversion and reversion rates of 11.6% and 24%, respectively, among HCWs in India, a high-incidence country [7].

We observed significant associations between the extent of baseline IFN-γ responses and the concordance of TST and QFT-GIT positivity, as well as the extent of TST induration and persistent QFT-GIT positivity. Accordingly, logistic regression analysis confirmed a significant relation between TST results, the extent of baseline IFN-γ responses and persistent QFT-GIT positivity. These findings are consistent with observations from several previous studies. In a British study following a TB outbreak, untreated contacts who remained persistently IGRA-positive had had TST reactions equivalent to Mantoux responses of 5-14 mm induration, whereas those with reversions had been TST-negative at baseline [13]. Two studies among Indian household contacts and HCWs, respectively, found that QFT-GIT reversions were significantly more likely when the baseline TST was negative and when the baseline IFN-γ response was close to the diagnostic threshold [7,12]. Likewise, a follow-up study among a U. S. foreign-born population found significantly lower baseline IFN-γ levels in individuals with reversions compared to those with persistently positive QFT-GIT results (mean 0.56 vs. 4.99 IU/ml) [25].

Contradictory results have been reported [34], but considering a recent systematic review, a previous TST may boost subsequent IFN-γ responses as the antigens ESAT-6 and CFP-10 are also present in PPD [35]. However, the lower frequency of positive QFT-GIT results at follow-up as well as the broad intervals between the TST applications and the subsequent tests indicate that boosting may not be a relevant phenomenon among our study population.

### Limitations

The present study is subject to limitations. Due to the small sample size, we were unable to determine independent risk factors for conversions and reversions. In addition, an inevitable limitation is the fact that there is no gold standard for the diagnosis of LTBI, and both IGRAs and the TST indicate lasting immune responses after exposure to MTB rather than the presence of viable replicating mycobacteria [2].

### Interpretation of findings

The fact that age and a positive prior TST were independent predictors of persistent QFT-GIT positivity may indicate the presence of long-standing MTB-specific immune responses, and thus, supports a rather remote immunological contact with MTB among the persistently QFT-GIT-positive individuals in our study [31,36]. No secondary TB cases were detected within the observational period. Recently, first evidence for the moderate predictive ability of a single positive IGRA result, or correspondingly, a documented IGRA conversion after recent extensive exposure to smear-positive TB regarding the progression to active TB was made available [37-41]. In these studies the rate of QFT-GIT-positive subjects progressing to active TB ranged from 2.8-17.2%. In contrast, up to date there is virtually no data regarding the interpretation and clinical relevance of persistently positive IFN-γ responses in serial testing or in the absence of recent extensive exposure [42,43]. However, our findings support the hypothesis that the risk of progression to active TB is likely to be low among those HCWs with persistently positive QFT-GIT results in the absence of recent extensive TB exposure.

Considering the dynamic characteristics of IFN-γ responses over time, which increase the chance of IGRA conversions and reversions, a simplistic dichotomous negative-to-positive definition may not be appropriate [44]. Although only a limited number of recent studies focused on the within-subject variability (reproducibility) of IGRA results [18-20,35], a variety of different borderline or uncertainty zones around the manufacturers' predefined cut-points as well as definitions of "true" conversions and reversions have been suggested in order to improve the interpretation of IGRA results in serial testing [7,9,12,18-20,25]. When we arbitrarily applied a borderline zone of 0.20-0.70 IU/ml [20], and an increase from < 0.35 to ≥ 0.70 IU/ml as a definition of a "true" conversion [7,12], only one of three conversions (1/162, 0.6%) and half of six reversions (3/18, 16.7%) may be considered as "true" conversion and reversion, respectively (Table 3). We observed three individuals with sharp positive-to-negative IFN-γ declines (No. 8, 72 and 95, compare Table 3), which may rather represent "true" reversions, e. g. due to clearing of acute infection or transition into dormancy than nonspecific variation [10,42,44].

### Clinical relevance of findings

Nonspecific variation, conversions, and reversions occur with IGRA serial testing, just as they do with the TST [7,35]. Our data suggests the usefulness of a borderline zone including unspecific variation around the manufacturer's predefined cut-off in order to avoid misinterpretations of IGRA results. Values within this zone should be interpreted with caution, and relevant clinical information should always be considered. One should bear in mind that neither LTBI nor active TB can be completely excluded by a single or even repeated negative IGRA results [10,45,46]. On the contrary, the possibility of a false positive IGRA result should be considered in TST-negative/IGRA-positive subjects, especially if it is close to the cut-off. Hence, laboratories should provide absolute IFN-γ values, and expert opinion should be sought for the interpretation of IGRA results in serial testing, if necessary.

We found that older HCWs, those with concordantly positive TST and QFT-GIT results, and those with high baseline IFN-γ responses had a significant chance to remain persistently QFT-GIT-positive over a median interval of 18 weeks. Consequently, repeated QFT-GIT testing may not be the diagnostic tool of choice in order to follow-up these subjects according to their workplace risk, and hence, a chest X-ray should be favored instead. However, these findings need further confirmation.

So far, it appears that IGRA responses over time significantly depend on the epidemiologic setting, in which these tests are applied, and different thresholds may be appropriate for different populations. However, uniform definitions of QFT-GIT conversions, reversions, and borderline zones among different populations remain to be defined yet. Thus, further research on the within-subject variability and the predictive value of (repeatedly positive) IGRA responses (and their predictors) in serial testing is warranted [10,35,43,44].

## Conclusions

The QFT-GIT's utility for the application in serial testing was limited by a substantial proportion of reversions. This shortcoming could be overcome by the implementation of a borderline zone for the interpretation of QFT-GIT results. However, further studies are needed to clearly define the within-subject variability of the QFT-GIT and to confirm that increasing age, concordantly positive TST results, and the extend of baseline IFN-γ responses may predict the persistence of QFT-GIT positivity over time in serially tested HCWs with only a low or medium TB screening risk in a TB low-incidence setting.

## Abbreviations

BCG: Bacillus Calmette-Guérin; CDC: Centers for Disease Control and Prevention; CI: confidence interval; ER: emergency room; F: female; HCWs: health care workers; ID: infectious diseases; IFN: interferon; IGRA: interferon-gamma release assay; LTBI: latent tuberculosis infection; M: male; MTB: Mycobacterium tuberculosis; OR: odds ratio; OSH: occupational safety and health; PPD: purified protein derivate; QFT-GIT: QuantiFERON^®^-TB Gold In-Tube; SNCP: smear-negative, culture-positive (tuberculosis source case); SPCP: smear-positive, culture-positive (tuberculosis source case); TB: tuberculosis; TST: tuberculin skin test; TU: tuberculin unit.

## Competing interests

The authors declare that they have no competing interests.

## Authors' contributions

FCR conceived and designed the study, performed the statistical analysis, interpreted the data, supervised the study, and drafted the manuscript. AN participated in the study design, data interpretation, statistical analysis, and revised the manuscript critically for important intellectual content. AS participated in the study design, and revised the manuscript critically for important intellectual content. SS participated in the study design, interviewed the HCW, applied and read the TST. GSW contributed to the study design, supervised the study, and revised the manuscript critically for important intellectual content. GR contributed to the study design, the analysis and interpretation of data, supervised the study, and revised the manuscript critically for important intellectual content. All authors read and approved the final manuscript.

## Authors' information

Part of the data was presented at the 18^th ^European Respiratory Society Annual Congress 2008 in Berlin, Germany [47] and at the 78^th ^Annual Congress 2010 of the Swiss Society of Internal Medicine in Basel, Switzerland [48].

## Pre-publication history

The pre-publication history for this paper can be accessed here:

http://www.biomedcentral.com/1471-2334/10/220/prepub

## Supplementary Material

Additional file 1 **Influence of exposure to TB source cases on subsequent test results**. The data demonstrates that there were no significant differences between the median cumulative exposure times with regard to subsequent test results among the subpopulation recruited from contact investigations, and furthermore, provides a detailed description of the four HCWs that had been exposed to smear-positive TB source cases. Click here for file

Additional file 2 **Table S2 - Agreement and time interval between prior and baseline TST and QFT-GIT results**. This table demonstrates that the prior TST had no significant effect on baseline TST and QFT-GIT results. Click here for file
