# Supplementary material for: Predictors of persistently positive Mycobacterium-tuberculosis-specific interferon-gamma responses in the serial testing of health care workers
Source: BMC Infect Dis. 2010 Jul 23;10:220. doi: 10.1186/1471-2334-10-220 (PMC2916913; doi:10.1186/1471-2334-10-220)
Supplement: Additional file 1 — Influence of exposure to TB source cases on subsequent test results. The data demonstrates that there were no significant differences between the median cumulative exposure times with regard to subsequent test results among the subpopulation recruited from contact investigations, and furthermore, provides a detailed description of the four HCWs that had been exposed to smear-positive TB source cases. [file 1471-2334-10-220-S1.PDF]

Predictors of persistently positive Mycobacterium-tuberculosis-specific interferon-gamma responses in the serial testing of health care workers

Felix C Ringshausen, Albert Nienhaus, Anja Schablon, Stephan Schlösser,

Gerhard Schultze-Werninghaus, Gernot Rohde

Additional file 1: Influence of exposure to TB source cases on subsequent test results

Table S1 - Influence of the cumulative exposure on subsequent test results

	Test result	n (%)	Median cumulative exposure time in min (range)	p-value for difference*
Baseline TST > 5mm (n = 134)	Positive<br>Negative	40 (29.9)<br>94 (70.1)	60 (5–2520)<br>83 (3–4000)	0.28
Baseline TST ≥ 10 mm (n = 134)	Positive<br>Negative	33 (24.6)<br>101 (75.4)	60 (5–2520)<br>60 (3–4000)	0.87
Baseline QFT-GIT# (n = 133)	Positive<br>Negative	17 (12.8)<br>116 (87.2)	60 (6–2625)<br>60 (3–4000)	0.49
Follow-up QFT-GIT# (n = 131)	Positive<br>Negative	12 (9.2)<br>119 (90.8)	38 (6–525)<br>60 (3–3308)	0.056

*Differences between the median cumulative exposure time were determined by the Mann-Whitney-U-test. #Individuals with indeterminate QFT-GIT results were excluded from this analysis. QFT-GIT = QuantiFERON®-TB Gold In-Tube. TST = tuberculin skin test.

As there were only four HCWs, who had been exposed to smear-positive TB source cases, a sound statistical analysis of the influence of the source cases' smear status on subsequent test results was unfortunately not possible. However, three of these four subjects, a 45-year old male, and a 47- and 55-year old female, respectively, had been exposed ≤ 1 hour and had had positive prior TST results 5 years ago in the median. They all had baseline TST results ≥ 10 mm induration and stable IFN-γ responses at baseline as well as follow-up resulting in persistently positive QFT-GIT results (12.80 and 2.40, 0.50 and 0.70, and 1.20 and 1.40 UI/ml, in the baseline and follow-up QFT-GIT, respectively). The other HCW, a 26-year old female, had been exposed to a smear positive source case for 8 hours, had not had a prior TST, had a negative baseline TST with an induration of 0 mm, a borderline positive baseline QFT-GIT result of 0.40 IU/ml, and a negative follow-up QFT-GIT result of 0.00 IU/ml.
