# Supplementary material for: Predictors of persistently positive Mycobacterium-tuberculosis-specific interferon-gamma responses in the serial testing of health care workers
Source: BMC Infect Dis. 2010 Jul 23;10:220. doi: 10.1186/1471-2334-10-220 (PMC2916913; doi:10.1186/1471-2334-10-220)
Supplement: Additional file 2 — Table S2 - Agreement and time interval between prior and baseline TST and QFT-GIT results. This table demonstrates that the prior TST had no significant effect on baseline TST and QFT-GIT results. [file 1471-2334-10-220-S2.PDF]

Predictors of persistently positive Mycobacterium-tuberculosis-specific interferon-gamma responses in the serial testing of health care workers

Felix C Ringshausen, Albert Nienhaus, Anja Schablon, Stephan Schlösser,
Gerhard Schultze-Werninghaus, Gernot Rohde

Additional file 2: Table S2 - Agreement and time interval between prior and baseline TST and QFT-GIT results

Table S2 - Agreement and time interval between prior and baseline TST and QFT-GIT results

	Prior TST (n = 129)				
Baseline TST > 5mm	Positive (n = 54)<br>n (%)	Negative (n = 75)<br>n (%)	Agreement	Median time interval between tests in months (range)	p-value for difference*
Positive (n = 39)	23 (17.8)	16 (12.4)	Raw = 63.5%	65 (3–454)	0.069
Negative (n = 90)	31 (24.0)	59 (45.7)	κ = 0.22#	56 (5–342)	
Baseline TST ≥ 10 mm					
Positive (n = 32)	19 (14.7)	13 (10.1)	Raw = 62.8%	61 (5–454)	0.057
Negative (n = 97)	35 (27.1)	62 (48.1)	κ = 0.19#	58 (5–342)	
Baseline QFT-GIT					
Positive (n = 14)	9 (7.0)	5 (3.9)	Raw = 61.2%	72 (5–454)	0.056
Negative (n = 115)	45 (34.9)	70 (54.3)	κ = 0.11§	58 (5–342)	

*Differences between the median time intervals between tests were determined by the Mann-Whitney-U-test. #p < 0.05, each. §p = 0.089. QFT-GIT = QuantiFERON®-TB Gold In-Tube. TST = tuberculin skin test.
